# Supplementary material for: Rapid response of arbuscular mycorrhizal fungal communities to short-term fertilization in an alpine grassland on the Qinghai-Tibet Plateau
Source: PeerJ. 2016 Jul 12;4:e2226. doi: 10.7717/peerj.2226 (PMC4950554; doi:10.7717/peerj.2226)
Supplement: Supplemental Information 1 [file peerj-04-2226-s001.doc]

Table S1: Summary of treatment groups of this study at QTP station.

| Treatments | Fertilizing amount |
| --- | --- |
| Control | **—** |
| N | 100 kg N ha^-1^yr^-1^ |
| P | 50 kg P ha^-1^yr^-1^ |
| NP | 100 kg N ha^-1^yr^-1^, 50 kg P ha^-1^yr^-1^ |

Table S2: Summary of the main biogeochemical variables. The values in parentheses represent the standard deviations. Superscript letters represent significant differences based on Tukey’s HSD comparisons (*P* < 0.05).

| Variables | Control | N | P | NP |
| --- | --- | --- | --- | --- |
| Soil pH | 7.32 (0.50)^a^ | 7.18 (0.63)^a^ | 7.67 (0.36)^a^ | 7.01 (0.62)^a^ |
| Soil moisture (%) | 26.5 (4.2)^a^ | 22.0 (4.8)^a^ | 23.3 (2.5)^a^ | 22.8 (3.3)^a^ |
| NO_3_^-^ (mg/kg) | 19.6 (0.6)^b^ | 26.3 (1.7)^a^ | 18.9 (2.8)^b^ | 27.3 (5.5)^a^ |
| NH_4_^+^ (mg/kg) | 9.16 (2.11)^a^ | 9.71 (1.42)^a^ | 12.22 (4.28)^a^ | 10.00 (5.17)^a^ |
| Total nitrogen (%) | 0.65 (0.03)^a^ | 0.61 (0.05)^a^ | 0.63 (0.05)^a^ | 0.64 (0.08)^a^ |
| Total carbon (%) | 6.49 (0.34)^a^ | 6.07 (0.55)^a^ | 6.28 (0.74)^a^ | 6.41 (1.02)^a^ |
| Total phosphorus (%) | 0.79 (0.01)^b^ | 0.82 (0.07)^b^ | 0.94 (0.08)^a^ | 0.90 (0.01)^ab^ |
| Dissolved organic N (mg/kg) | 31.3 (4.1)^b^ | 41.7 (5.1)^a^ | 32.8 (7.4)^b^ | 46.3 (10.1)^a^ |
| Dissolved organic C (mg/kg) | 178.7 (18.6)^a^ | 195.8 (37.3)^a^ | 170.7 (14.4)^a^ | 180.9 (29.9)^a^ |
| Available phosphorus (mg/kg) | 6.20 (1.54)^b^ | 4.86 (2.00)^b^ | 36.3 (12.7)^a^ | 46.2 (4.16)^a^ |
| Root biomass (kg/m^2^) | 2.06 (0.46)^a^ | 2.06 (0.57)^a^ | 2.25 (0.51)^a^ | 2.33 (0.46)^a^ |
| Graminoid biomass (g/m^2^) | 233 (17)^b^ | 265 (77)^ab^ | 272 (67)^ab^ | 414 (130)^a^ |
| Sedge biomass (g/m^2^) | 10.9 (5.0)^ab^ | 27.6 (15.4)^a^ | 7.2 (4.5)^b^ | 1.7 (0.6)^b^ |
| Legume biomass (g/m^2^) | 21.2 (6.9)^ab^ | 12.0 (4.3)^b^ | 28.1 (9.1)^a^ | 4.4 (3.6)^c^ |
| Forbs biomass (g/m^2^) | 57 (17)^b^ | 106 (21)^b^ | 102 (61)^b^ | 247 (98)^a^ |
| Total aboveground biomass (g/m^2^) | 322 (21)^b^ | 411 (81)^b^ | 409 (94)^b^ | 667 (57)^a^ |

Table S3: Pearson correlations (*r*) between AMF alpha-diversity and biogeochemical properties. OTU: operational taxonomic unit; PD: phylogenetic diversity; SM: soil moisture; DON: dissolved organic nitrogen; DOC: dissolved organic carbon; TC: total carbon; TN: total nitrogen; TP: total phosphorus; AP: available phosphorus; RB: root biomass; TB: total aboveground biomass. *: *P* < 0.05; **: *P* < 0.01.

|  | Alpha-diversity | |
| --- | --- | --- |
|  | OTU | PD |
| pH | -0.120 | -0.146 |
| SM | 0.104 | 0.012 |
| NO_3_^-^ | **0.457*** | **0.489*** |
| NH_4_^+^ | 0.332 | 0.002 |
| DON | **0.549*** | **0.494*** |
| DOC | 0.131 | 0.172 |
| TN | 0.196 | -0.006 |
| TC | 0.213 | 0.004 |
| TP | 0.180 | 0.062 |
| AP | **0.543*** | **0.518*** |
| RB | 0.371 | 0.376 |
| Gramineae | **0.498*** | **0.510*** |
| Sedge | -0.038 | -0.288 |
| Legume | **-0.608**** | **-0.757**** |
| Forbs | **0.564*** | **0.668**** |
| TB | **0.631**** | **0.668**** |

Table S4: Differences in AMF community composition across treatments as determined by Analyses of Similarities (ANOSIM).

|  | ANOSIM | |
| --- | --- | --- |
|  | *r* | *P* |
| Control vs N | **0.330** | **0.017** |
| Control vs P | **0.436** | **0.012** |
| Control vs NP | **0.864** | **0.008** |
| N vs P | 0.250 | 0.073 |
| N vs NP | **0.764** | **0.013** |
| P vs NP | 0.180 | 0.080 |

Table S5: Correlation coefficients (*r*) and significance (*P*) were determined by Mantel tests: comparing differences between samples in AMF community composition to differences between samples in biogeochemical variables. SM: soil moisture; DON: dissolved organic nitrogen; DOC: dissolved organic carbon; TC: total carbon; TN: total nitrogen; TP: total phosphorus; AP: available phosphorus; RB: root biomass; TB: total aboveground biomass.

| Variables | Mantel test | |
| --- | --- | --- |
|  | *r* | *P* |
| pH | 0.007 | 0.443 |
| SM | 0.014 | 0.357 |
| NO_3_^-^ | **0.199** | **0.045** |
| NH_4_^+^ | -0.141 | 0.862 |
| DON | 0.028 | 0.406 |
| DOC | 0.021 | 0.413 |
| TN | 0.060 | 0.303 |
| TC | 0.058 | 0.326 |
| TP | **0.218** | **0.012** |
| AP | **0.398** | **0.002** |
| RB | 0.042 | 0.323 |
| Gramineae | **0.251** | **0.043** |
| Sedge | -0.148 | 0.895 |
| Legume | **0.327** | **0.004** |
| Forbs | **0.259** | **0.024** |
| TB | **0.330** | **0.005** |

Table S6: Multiply regression tree (MRT) analysis of AMF community structure with environmental variables. The tree was done using “1se” method with 1,000 cross validations. AP: available phosphorus; TB: total aboveground biomass.

| Variables | MRT explained (%) |
| --- | --- |
| AP | **20.5** |
| TB | **8.4** |
| Legume | **7.1** |
| Gramineae | **1.0** |
| Total | **37.0** |

Table S7: Pearson correlations (*r*) between relative abundance of dominant AMF taxa and biogeochemical characteristics. SM: soil moisture; DON: dissolved organic nitrogen; DOC: dissolved organic carbon; TC: total carbon; TN: total nitrogen; TP: total phosphorus; AP: available phosphorus; RB: root biomass; TB: total aboveground biomass. *: *P* < 0.05; **: *P* < 0.01.

|  | *Claroideoglomeraceae* | *Gigasporaceae* | *Glomeraceae* | *Diversisporaceae* | *Acaulosporaceae* |
| --- | --- | --- | --- | --- | --- |
| pH | 0.122 | 0.119 | -0.424 | 0.132 | -0.087 |
| SM | 0.157 | 0.024 | 0.141 | 0.047 | 0.025 |
| NO_3_^-^ | 0.058 | 0.025 | 0.264 | -0.063 | -0.323 |
| NH_4_^+^ | 0.236 | -0.218 | 0.084 | 0.071 | -0.260 |
| DON | 0.157 | -0.133 | **0.470*** | -0.040 | **-0.459*** |
| DOC | -0.281 | 0.288 | 0.109 | -0.149 | 0.097 |
| TN | 0.266 | -0.265 | -0.058 | -0.292 | 0.157 |
| TC | 0.246 | -0.255 | -0.022 | -0.271 | 0.134 |
| TP | 0.414 | **-0.445*** | 0.181 | 0.192 | -0.441 |
| AP | **0.567**** | **-0.625**** | 0.375 | 0.219 | **-0.673**** |
| RB | 0.079 | 0.071 | 0.070 | 0.186 | -0.418 |
| Gramineae | 0.312 | -0.433 | 0.137 | 0.395 | -0.440 |
| Sedge | -0.327 | 0.340 | -0.117 | 0.019 | 0.121 |
| Legume | 0.334 | -0.199 | **-0.518*** | -0.244 | 0.295 |
| Forbs | 0.485 | -0.410 | **0.520*** | -0.226 | **-0.538*** |
| TB | **0.515*** | **-0.543*** | 0.367 | 0.122 | **-0.607*** |


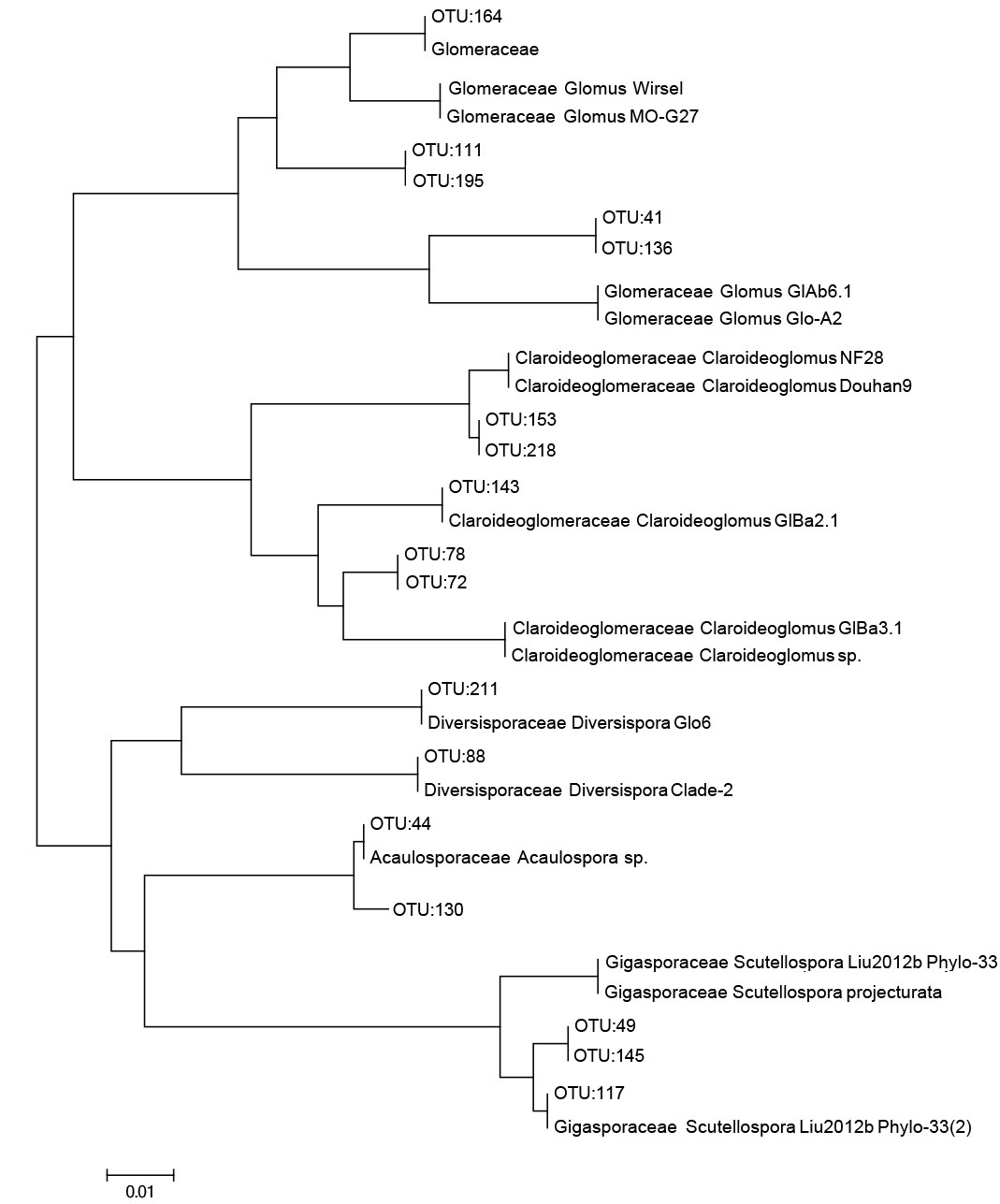
Figure S1: Phylogenetic tree of dominant AMF OTUs (the relative abundance > 1%) at QTP station based on neighbor-joining method.


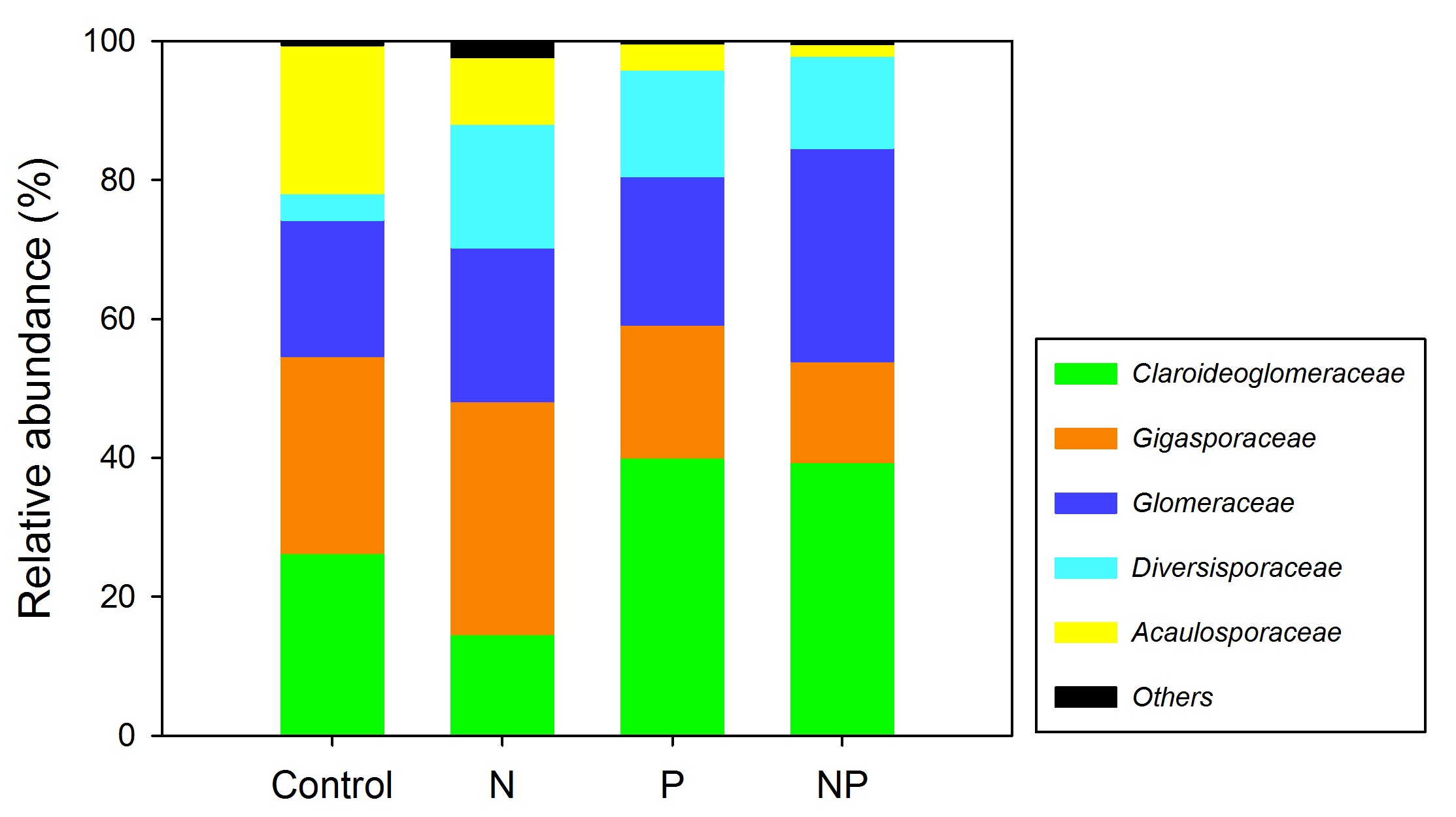
Figure S2: Relative abundances of the dominant AMF families across treatment groups at QTP station.
